# Obesity-induced chronic inflammation in high fat diet challenged C57BL/6J mice is associated with acceleration of age-dependent renal amyloidosis

**DOI:** 10.1038/srep16474

**Published:** 2015-11-13

**Authors:** Roel A. van der Heijden, Johan Bijzet, Wouter C. Meijers, Gopala K. Yakala, Robert Kleemann, Tri Q. Nguyen, Rudolf A. de Boer, Casper G. Schalkwijk, Bouke P. C. Hazenberg, Uwe J. F. Tietge, Peter Heeringa

**Affiliations:** 1 Department of Pathology & Medical Biology, Medical Biology Section, University Medical Center Groningen, University of Groningen, Groningen, The Netherlands; 2 Department of Rheumatology and Clinical Immunology, University Medical Center Groningen, University of Groningen, Groningen, The Netherlands; 3 Department of Cardiology, University Medical Center Groningen, University of Groningen, Groningen, The Netherlands; 4 Translational Laboratory in Genetic Medicine (TLGM), Agency for Science, Technology and Research (A*STAR) and Department of Medicine, National University of Singapore, Singapore; 5 Department of Metabolic Health Research, The Netherlands Organization for Applied Scientific Research (TNO), Leiden, The Netherlands; 6 Department of Pathology, University Medical Center Utrecht, Utrecht, The Netherlands; 7 Experimental Medicine, Maastricht University Medical Centre, Maastricht, The Netherlands; 8 Department of Pediatrics, University Medical Center Groningen, University of Groningen, Groningen, The Netherlands; 9 Top Institute Food and Nutrition, Wageningen, The Netherlands

## Abstract

Obesity-induced inflammation presumably accelerates the development of chronic kidney diseases. However, little is known about the sequence of these inflammatory events and their contribution to renal pathology. We investigated the effects of obesity on the evolution of age-dependent renal complications in mice in conjunction with the development of renal and systemic low-grade inflammation (LGI). C57BL/6J mice susceptible to develop age-dependent sclerotic pathologies with amyloid features in the kidney, were fed low (10% lard) or high-fat diets (45% lard) for 24, 40 and 52 weeks. HFD-feeding induced overt adiposity, altered lipid and insulin homeostasis, increased systemic LGI and adipokine release. HFD-feeding also caused renal upregulation of pro-inflammatory genes, infiltrating macrophages, collagen I protein, increased urinary albumin and NGAL levels. HFD-feeding severely aggravated age-dependent structural changes in the kidney. Remarkably, enhanced amyloid deposition rather than sclerosis was observed. The degree of amyloidosis correlated significantly with body weight. Amyloid deposits stained positive for serum amyloid A (SAA) whose plasma levels were chronically elevated in HFD mice. Our data indicate obesity-induced chronic inflammation as a risk factor for the acceleration of age-dependent renal amyloidosis and functional impairment in mice, and suggest that obesity-enhanced chronic secretion of SAA may be the driving factor behind this process.

Renal aging is characterized by various structural changes that manifest as glomerulosclerosis (GS), tubular atrophy and interstitial fibrosis^1^. These age-related changes ultimately lead to a decline in renal function^2^ and may result in chronic kidney disease (CKD). As life expectancy continues to increase, the prevalence of CKD in the aging population is likely to increase as well^3^,^4^. Besides age, environment and lifestyle are important risk factors for CKD. More specifically, the alarming increase in (morbid) obesity, resulting from our changing dietary habits^5^ and sedentary lifestyles^6^, has been shown to increase the incidence of CKD^7^,^8^. Although the development and progression of CKD is clearly multifactorial, compelling evidence points towards low-grade inflammation as a driving force^9^,^10^. Whereas aging and obesity are both associated with a state of chronic inflammation^11^,^12^, obesity-induced chronic inflammation may be established much earlier in life^13^ and hence its detrimental effects are induced earlier and are longer lasting than those observed with aging.

The exact mechanisms by which chronic inflammation contributes to CKD remain to be elucidated. However, increasing evidence supports a crucial role of renal macrophages. As macrophages infiltrate and accumulate in the interstitium, pro-fibrotic factors are released locally, promoting the deposition of extracellular matrix proteins such as collagen type I eventually leading to structural changes^14^. These structural changes are associated with impaired vaso-contractility and disturbance of the filtrating capacity of the kidney^15^.

The evolution of obesity-associated CKD in humans is difficult to study since clinical disease hallmarks, such as increased albuminuria and decreased eGFR, arise at a late stage, often when irreversible organ impairment has already taken place. Therefore, from a diagnostic as well as therapeutic perspective, disseminating the various stages of disease development is crucial.

The aim of the current study was to provide insight into the onset and evolution of both obesity- and age-associated renal pathologies and how these pathologies manifest at the functional and systemic level. We hypothesized that a chronic, obesity-enhanced elevation of systemic pro-inflammatory factors would accelerate the appearance and progression of age-associated renal dysfunction. To address this hypothesis, in C57BL/6j mice prone to develop age-dependent renal pathologies^19^ we established and monitored high fat diet-induced obesity (DIO) for a period of 52 weeks, compared the structural and functional alterations in the kidneys to age-matched low fat diet controls and linked these alterations to local and systemic inflammatory parameters.

## Results

### HFD-feeding leads to adiposity and metabolic dysfunction

**Figure 1:**
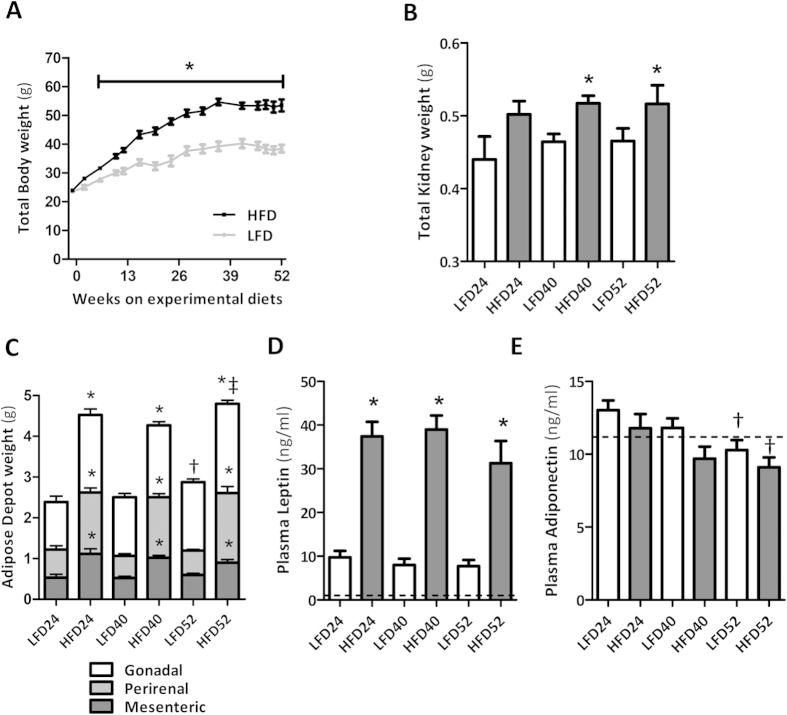
Weight gain and adiposity with aging and HFD-feeding. (**A**) Gradual age-associated increase in body weight (23.9 ± 0.2 g at baseline), (**B**) total kidney weight and (**C**) adipose depot weights were enhanced by prolonged HFD-feeding. (**D**) Adiposity was further reflected by an increase in plasma leptin. (**E**) Decrease in plasma adiponectin was observed with aging but did not reach significance between LFD and HFD. Data are expressed as mean ± SEM. Dotted lines represent baseline values for leptin (0.13 ± 0.05 ng/ml) and adiponectin (11.18 ± 0.65 ng/ml). Significance level set as p < 0.05. *significantly different from LFD same time point, ^†^significantly different from same diet 24 w, ^‡^significantly different from same diet at 40 w.

In low fat diet (LFD) and high fat diet (HFD) mice, body weight increased gradually over time, reaching a plateau at 36 weeks. The average body weight in HFD mice was significantly higher than in LFD mice from 6 weeks onwards (Fig. 1A). All HFD mice showed a marked increase in kidney weight and adipose tissue mass for gonadal, perirenal and mesenteric depots analyzed at all time points as compared to LFD mice (Fig. 1B,C). In HFD mice plasma levels of leptin were strongly increased at all three time points when compared to LFD mice (Fig. 1D). Adiponectin, a factor known to be inversely correlated with obesity, gradually decreased in HFD mice, but no significant difference with LFD mice was reached (p = 0.07 at 40 w; Fig. 1E).

**Figure 2:**
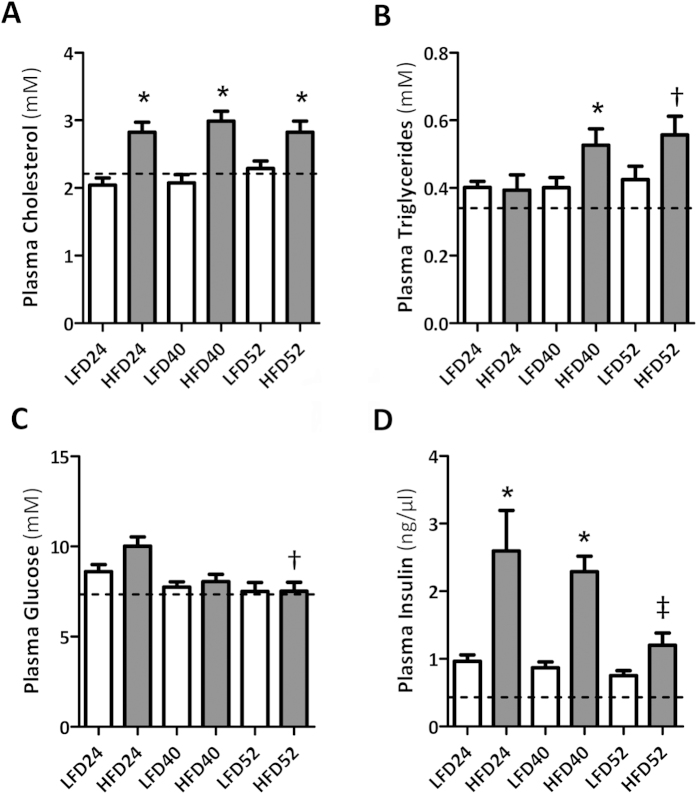
Metabolic dysfunction with aging and HFD feeding. (**A**) HFD-feeding leads to hyperlipidemia as evidenced by a significant increase in plasma cholesterol and (**B**) plasma triglyceride levels. (**C**) Blood glucose levels did not change over time and were not different between LFD and HFD mice, but (**D**) were paralleled by elevated plasma insulin levels in HFD mice. Data are expressed as mean ± SEM. Dotted lines represent baseline values for cholesterol (2.21 ± 0.06 mM), triglycerides (0.34 ± 0.03 mM), glucose (7.34 ± 0.32 mM) and insulin (0.43 ± 0.08 ng/μl). Significance level set at p < 0.05. *significantly different from LFD same time point, ^†^significantly different from same diet 24 w, ^‡^significantly different from same diet 40 w.

Adiposity is associated with the release of excess lipids into the circulation resulting in systemic hyperlipidemia. In HFD mice, plasma cholesterol was significantly increased at all time points compared to LFD mice (Fig. 2A), whereas elevated plasma triglyceride levels were detected at 40 w (Fig. 2B). HFD-increased insulin levels were detected but no significant changes in plasma glucose levels were observed throughout the experimental period (Fig. 2C,D).

### HFD-induced obesity aggravates age-dependent albuminuria and enhances urinary NGAL levels

**Figure 3:**
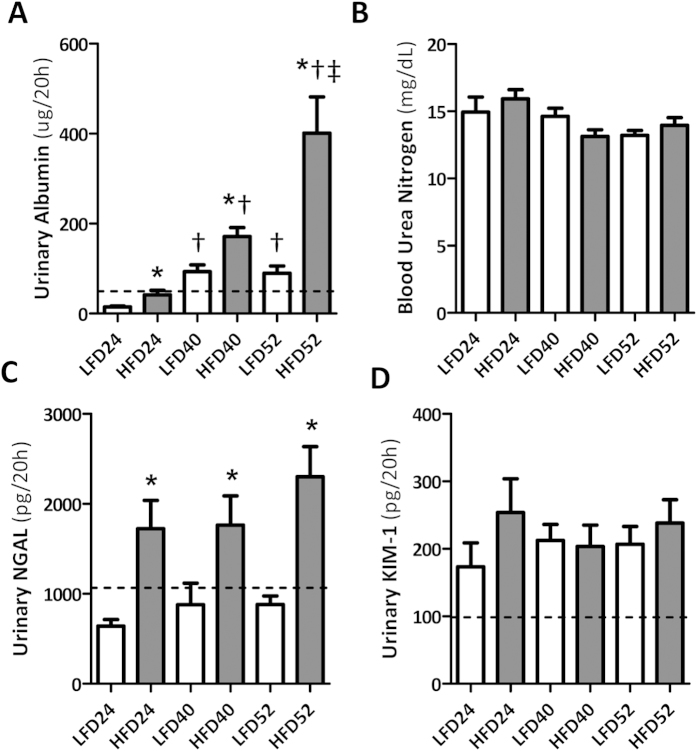
Renal (dys)function and injury markers with aging and HFD-feeding. (**A**) With aging a significant increase in albuminuria was observed which was enhanced by HFD feeding. (**B**) Plasma blood urea nitrogen levels did not change. (**C**) NGAL levels showed no age-associated changes but were significantly increased in HFD urine samples for all time points. (**D**) Urinary KIM-1 levels showed no significant changes. Data expressed as mean ± SEM. Dotted lines represent baseline values for albumin (49.86 ± 4.67), NGAL (1086.00 ± 47.63) and KIM-1 (98.37 ± 6.175). Significance level set at p < 0.05. *significantly different from LFD at same time point, ^†^significantly different from same diet 24 w, ^‡^significantly different from same diet 40 w.

We observed an age-dependent increase in albuminuria, which was significantly aggravated by HFD (Fig. 3A). Blood urea nitrogen (BUN) levels did not show age-dependent alterations nor differences between dietary groups (Fig. 3B). Interestingly, neutrophil gelatinase-associated lipocalin (NGAL) levels, a protein secreted upon kidney injury, were already significantly higher in urine samples of HFD mice compared to LFD mice from 24 w onwards, but did not change over time (Fig. 3C). In contrast, urinary levels of kidney injury molecule 1 (KIM-1), a marker for kidney repair upon injury, did not differ between the diet groups, nor did they change over time (Fig. 3D).

Since increased blood pressure (BP) is a main driver of renal injury and dysfunction, BP was measured invasively. No significant increases in central BP were observed at any time point in HFD mice compared to LFD mice (Suppl. 1A). Interestingly, despite the absence of hypertension, mild left ventricular hypertrophy did occur (Suppl. 1B), with an increase in various early-onset tissue markers for left ventricular hypertrophy (Suppl. 1D–F), albeit with normal systolic cardiac performance (Suppl. 1C).

### Age-dependent renal sclerosis is aggravated by HFD-induced obesity and displays features of amyloidosis

**Figure 4:**
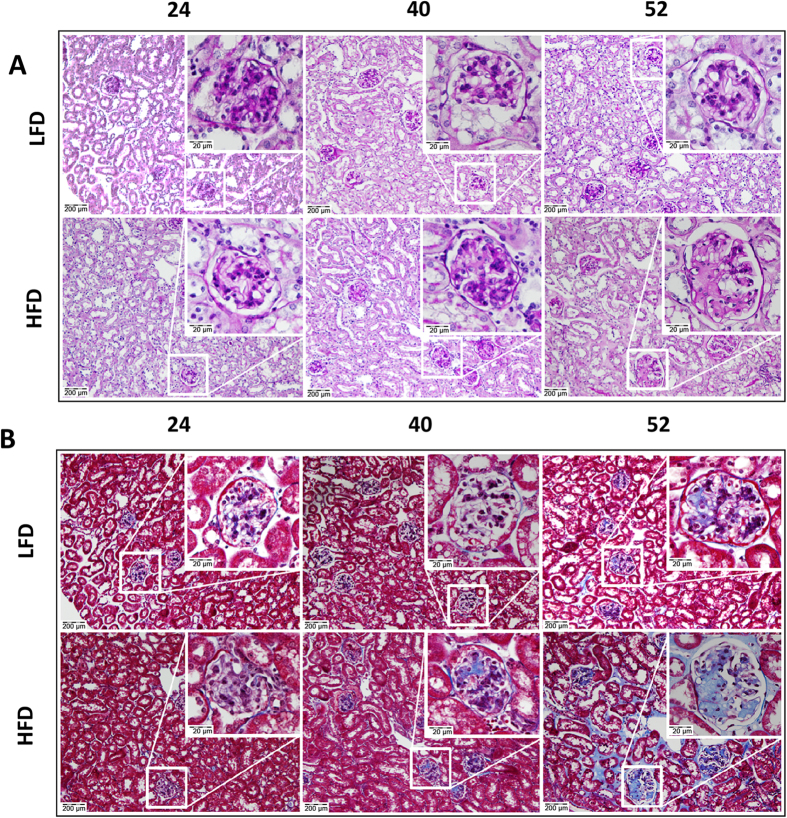
Development of renal sclerosis with aging and HFD-feeding. Representative histological pictures of the renal cortex (100×) and a magnified glomerulus (inset; 400×) of mice sacrificed after 24, 40 and 52 weeks of LFD (upper panels) versus HFD feeding (lower panels). (**A**,**B**) Consecutive tissue sections stained with periodic acid Schiff’s and Masson’s trichrome demonstrate first nodular appearance of age-associated sclerosis at 52 w in glomeruli of LFD fed mice. These nodules are advanced in HFD fed mice, being observed as early as 40 w, and become severely aggravated at 52 w, also affecting the tubulo-interstitial area.

**Figure 5:**
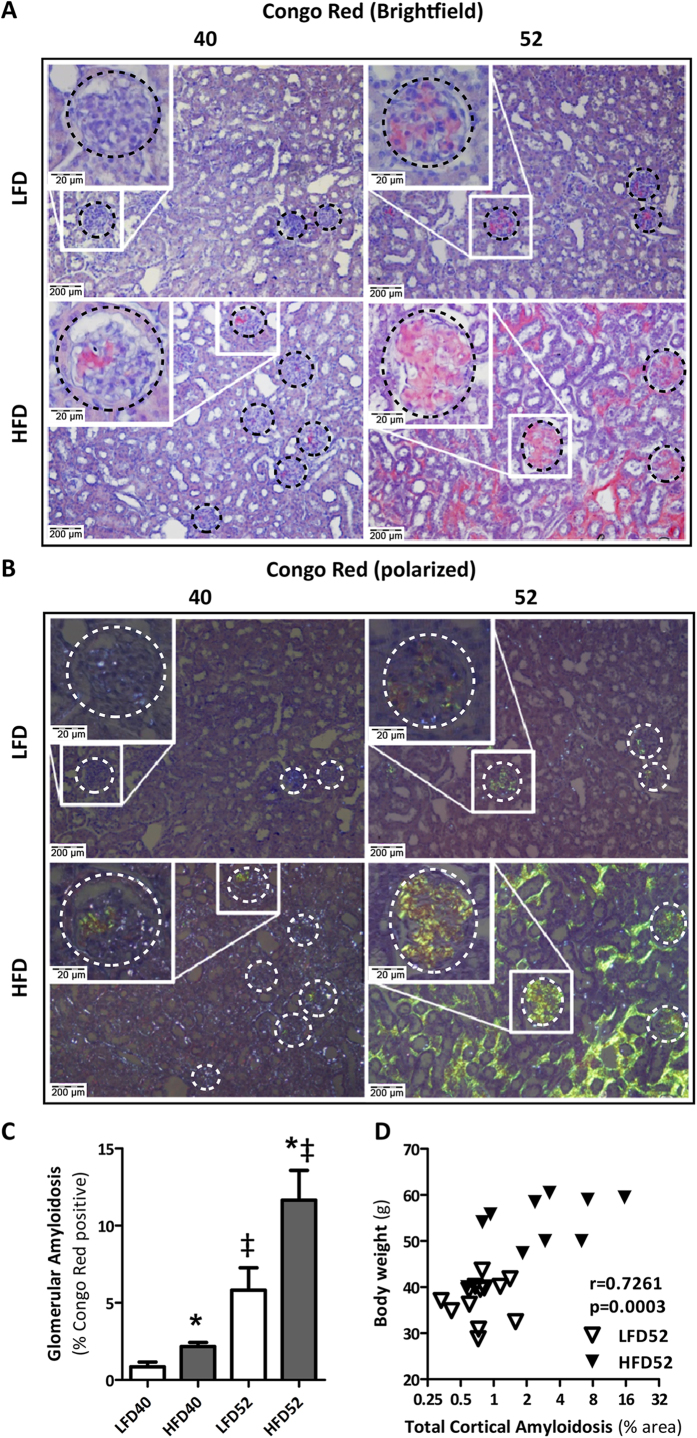
Renal amyloid deposits with aging and HFD-feeding. (**A**) Representative pictures of renal LFD and HFD sections stained for Congo Red and (**B**) the same sections assessed under polarized light show sclerotic lesions in the renal cortex, both interstitial (100×) and glomerular (insets; 400×). (**A**,**B**) Amyloid first appears in glomerular regions (indicated by dotted circles) at HFD40 and extensively affects tubulo-interstitium in HFD52. (**C**) Quantification of amyloidosis in glomeruli demonstrating that HFD mice are significantly more affected at all time points. Body weight and quantified amount of amyloid show a strong positive correlation (**C**; r = 0.7419; p = 0.002). Data expressed as mean ± SEM. Significance level set at p < 0.05. *significantly different from LFD at same time point, ^†^significantly different from same diet 24 w, ^‡^significantly different from same diet 40 w.

Histological analysis of renal tissue using Periodic Acid Schiff’s (PAS) and Masson-trichrome (MT) stainings revealed an age-associated, progressive sclerotic phenotype. Whereas the sclerotic areas in the LFD group first appeared focally in glomeruli at 52 w, the HFD group already exhibited these foci at 40 w and displayed markedly increased lesions at 52 w. At this time point, not only glomeruli were affected, but lesions were also observed in the tubulo-interstitium (Fig. 4A,B). To confirm the fibrotic nature of the observed lesions we quantified collagen I protein, the main form of collagen found in mature renal fibrotic lesions, by immunohistochemistry. At 24 w and 52 w a significant difference in the extent of collagen deposition was observed between LFD and HFD challenged mice. However, the extent of collagen deposition in HFD mice did not increase proportionally to the extent of the lesions as assessed by MT staining (Suppl. 2A,B). This finding, combined with the weak PAS staining of the lesions (Fig. 4A), prompted us to further investigate the nature of the amorphous material. We explored misfolded amyloidogenic protein aggregates as potential candidates. Staining with Congo Red (CR) in combination with microscopical assessment by polarized light, revealed the typical apple-green birefringence of the CR dye to be present in glomeruli as well as in the tubulo-interstitium (Fig. 5A,B). Quantification of CR staining in the renal cortex showed a significant increase of amyloid deposits in both LFD and HFD mice with aging which was significantly greater in HFD-treated mice (Fig. 5C). There was a strong correlation between total body weight and the extent of amyloidosis (Fig. 5D), suggesting a link with obesity. To determine whether the observed amyloidosis was organ specific or systemic, other organs were assessed by CR as well demonstrating amyloid deposits in small intestine, left ventricle, lung, adipose and hepatic tissues, particularly affecting the vasculature (Suppl. 3A–F).

### HFD-induced obesity results in chronic low-grade inflammation

**Figure 6:**
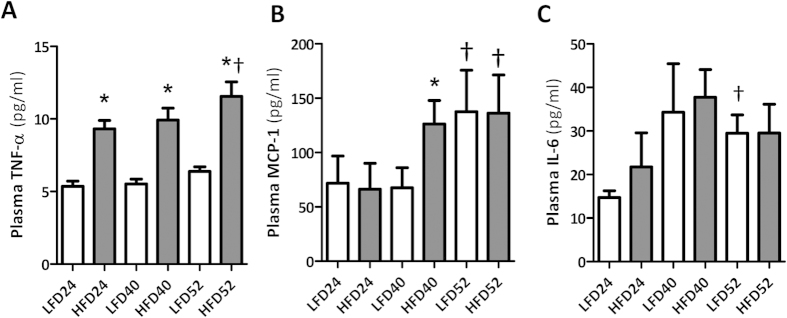
Systemic pro-inflammatory cytokine levels with aging and HFD-feeding. (**A**) Plasma levels of TNF-α are significantly higher in HFD mice at all time points and significantly increase in time for HFD mice. (**B**,**C**) MCP-1 and IL-6 levels show an age-associated increase which is advanced by HFD-feeding. Data expressed as mean ± SEM. Significance level set at p < 0.05. *significantly different from LFD at same time point, ^†^significantly different from same diet 24 w, ^‡^significantly different from same diet 40 w.

Since inflammation is considered a key contributor to the onset and progression of obesity-induced chronic renal disorders, we examined the sequence of inflammatory events and their temporal relationship with the observed pathological alterations in the kidney. In HFD mice, plasma levels of tumor necrosis factor alpha (TNF-α) were elevated from 24 w onwards. Monocyte chemoattractant protein 1 (MCP-1) levels increased significantly with aging in both LFD and HFD groups, but this increase became evident earlier (40 w) in HFD mice. Circulating interleukin 6 (IL-6) levels were not significantly increased in LFD mice and HFD mice over time relative to 24 w, except for the 52 w time point in LFD mice (Fig. 6A–C). Plasma levels of IL-1β were under the detection limit in all mice (data not shown).

**Figure 7:**
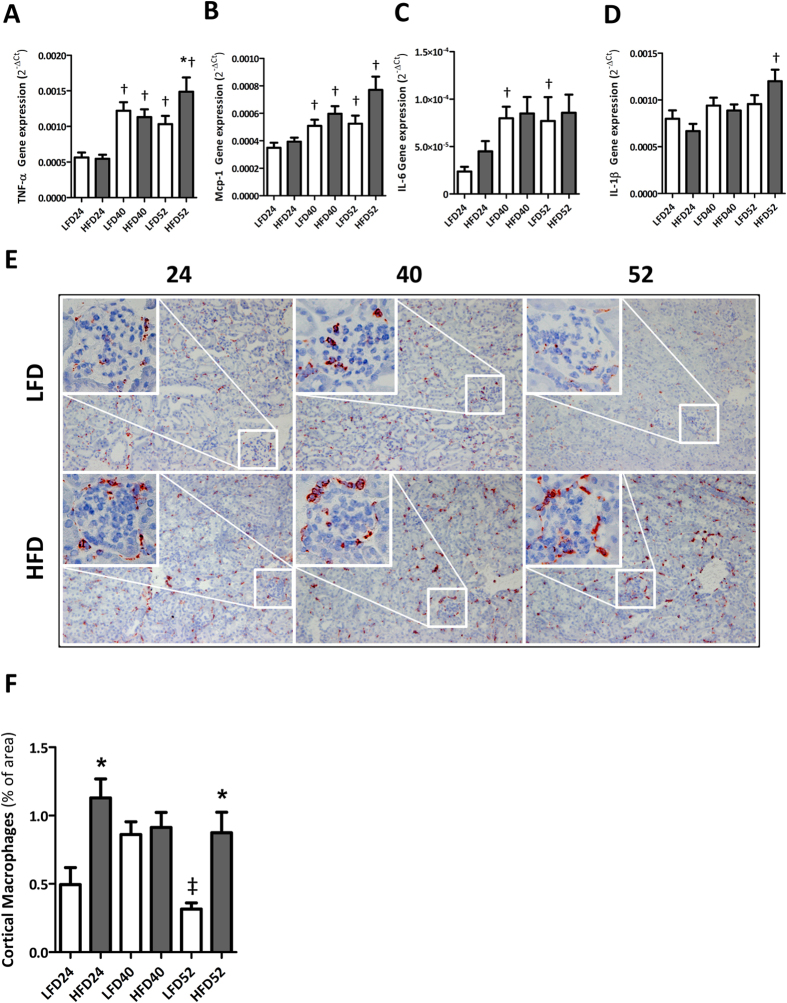
Renal inflammation with aging and HFD-feeding. (**A**,**D**) Renal gene expression analyses show an age-associated increase of TNF-α, MCP-1, IL-6 and IL-1β mRNA levels, significantly different between LFD and HFD for TNF-α and MCP-1 at 52 w. (**D**,**E**) Representative pictures of CD68+ positive macrophages in renal cortex (100×) and glomerulus (inset; 400×) at different time points showing significant differences between LFD and HFD at 24 w and 52 w. Data expressed as mean ± SEM. Significance level set at p < 0.05. *significantly different from LFD at same time point, ^†^significantly different from same diet 24 w, ^‡^significantly different from same diet 40 w.

Renal gene expression analyses revealed a significant age-dependent upregulation of TNF-α, MCP-1 and IL-6 mRNA of which TNF-α and MCP-1 were significantly increased in the HFD group at 52 weeks when compared to LFD mice (Fig. 7A–C). Moreover, renal mRNA expression levels of IL-1β in HFD mice increased significantly over time (Fig. 7D).

By immunohistochemistry, infiltration of CD68+ macrophages in glomeruli and tubulo-interstitium was observed in both LFD and HFD mice. The number of infiltrating macrophages in HFD mice was significantly increased at 24 w and 52 w when compared to LFD mice as quantified by morphometry (Fig. 7E,F). Moreover, CD68+ macrophage accumulation positively correlated with the extent of collagen I deposits (r = 0.795, p < 0.001) as assessed in matched cortical regions.

### HFD-induced elevation of systemic SAA levels is associated with enhanced SAA amyloid deposition

**Figure 8:**
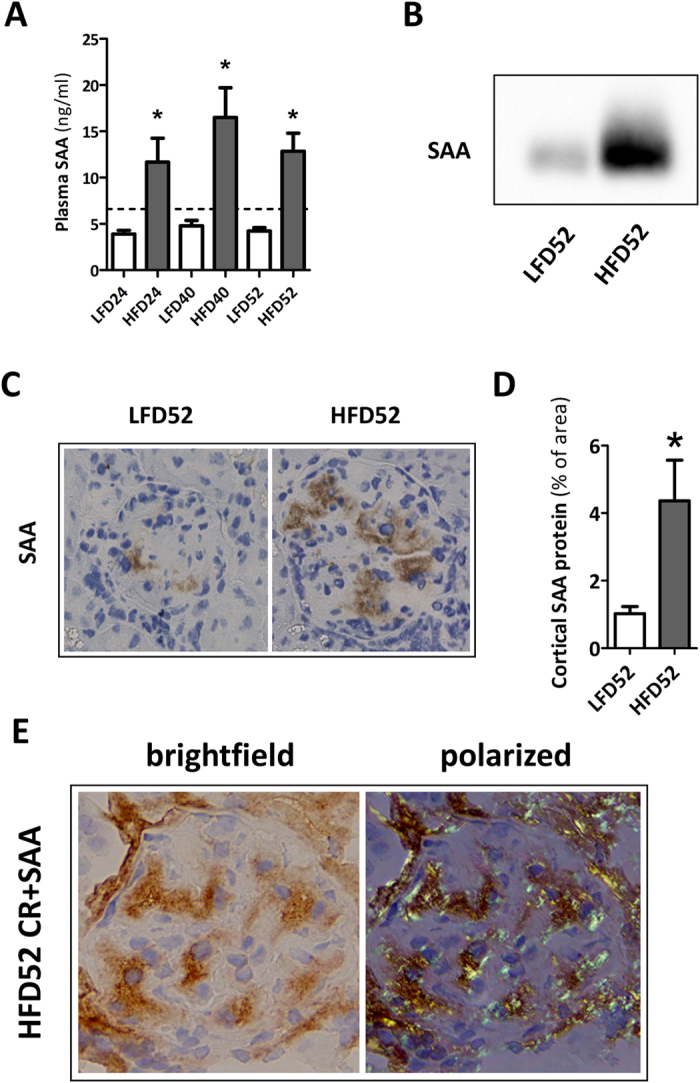
Plasma SAA concentrations and renal SAA deposition. (**A**) Plasma concentrations of SAA protein were significantly elevated in HFD mice at all time points. (**B**) Quantification of SAA in HDL showed more SAA present in HDL^HFD^ compared to HDL^LFD^ at 52 w. (**C**,**D**) Representative renal sections stained with SAA antibody show distinct localization of SAA protein in the glomerulus which is more abundant in HFD mice. (**E**) Congo red and SAA double staining confirmed SAA protein and amyloid to localize in the same region. Data expressed as mean ± SEM. Dotted lines represent baseline value for SAA (7.33 ± 1.24 ng/ml). Significance level set at p < 0.05. *significantly different from LFD same time point.

Amyloidosis is caused by the extracellular deposition of protein fibrils and many amyloidogenic proteins have been identified. However, irrespective of the origin of the amyloidogenic protein, all forms of amyloidosis share common histological features^16^. In AA amyloidosis the amyloid fibrils are derived from SAA, an acute phase protein produced by hepatocytes under inflammatory conditions^17^. Since obesity induced a state of low grade chronic inflammation, we postulated that the observed renal amyloidosis in our study originated from SAA deposits due to chronically elevated levels of SAA. Systemic SAA levels were significantly higher in HFD mice during the entire investigational period (Fig. 8A).

Besides its free form, SAA is predominantly found associated with high-density lipoproteins (HDL)^18^. Examination of the presence of SAA in the HDL fractions of pooled plasma samples showed HDL^HFD^ to contain a higher amount of SAA than HDL^LFD^ (Fig. 8B). In addition, by immunohistochemistry, positive staining for SAA in glomeruli and tubulo-interstitium (not shown) was observed in both LFD and HFD mice (Fig. 8C) which was significantly more extensive in HFD mice (Fig. 8D). Finally, combining CR with SAA staining, a strong co-localization of amyloid deposits and SAA was observed (Fig. 8E).

## Discussion

The current study was designed to provide insight into the evolution of both obesity- and age-associated renal pathologies. In C57BL/6J mice fed a LFD or a HFD, we investigated when and how renal pathology manifests at the functional and systemic level and how this associates with obesity and age-dependent inflammation. We demonstrate a gradual, age-dependent occurrence of mild albuminuria, tubulo-interstitial and glomerular structural changes, and increased expression of inflammatory mediators. When superimposed with a HFD, albuminuria and renal inflammation were detected earlier and increased in extent. Most strikingly, compared to LFD mice, HFD mice developed severely aggravated structural and morphological changes that showed features typical of amyloidosis.

Age-associated glomerulosclerosis with amyloid characteristics and ectopic lipid depositions was previously described in C57BL/6J mice^19^. Our study extends these observations by disseminating the development of this age-related phenotype over time and analyzing its association with the development of albuminuria and renal inflammatory parameters. More importantly, our study allowed examination of the detrimental effects caused by prolonged obesity superimposed to aging. Obesity has been shown to advance the appearance of many age-associated diseases including CKD^20^,^21^. Hence it is often referred to as an accelerating factor of the aging process^22^. Inflammation is known to negatively influence many age-associated processes, hence the systemic inflammation that accompanies obesity has been considered a driving factor behind this accelerated aging phenotype^23^. Indeed, our data demonstrate that obesity, when superimposed to aging, results in enhanced renal inflammation and accelerates and aggravates the functional and structural abnormalities in the kidneys as evidenced by increased albuminuria and extensive glomerular and tubulo-interstitial lesions in mice fed a HFD.

The enhanced renal inflammatory phenotype observed with obesity may have multiple origins. First, because the lipid storing capacity of the adipose tissue (AT) and liver is exceeded with obesity, lipid uptake from the circulation is impaired resulting in hyperlipidemia^24^,^25^. Consequently, lipid uptake in the kidneys may be enhanced causing lipotoxicity and inflammation^26^. Second, obesity induced by HFD feeding may cause insulin resistance (IR) leading to systemic hyperglycemia which is known to be associated with endothelial dysfunction and enhanced renal inflammation^27^,^28^. Furthermore, prolonged hyperglycemia is known to provoke hypertension^29^,^30^, which in the kidney glomerulus can lead to hyperfiltration and inflammation. Whereas in our study mice did not develop hyperglycemia or signs of hypertension, we did observe a rise in plasma lipids. This was evident as illustrated by elevated plasma cholesterol levels in HFD mice from 24 weeks onwards and elevated triglycerides levels from 40 w onwards. The latter was paralleled by glomerular lipid accumulation (data not shown). These data suggest that the enhanced inflammatory phenotype in the HFD mice is likely linked to hyperlipidemia.

Previously, we have reported that excessive adiposity is associated with AT inflammation^31^. Prolonged AT inflammation has been shown to enhance systemic release of pro-inflammatory cytokines such as TNF-α, IL-6 and IL-1β^12^ that may cause endothelial activation thereby promoting leukocyte recruitment in the kidney. In the current study we particularly detected increased circulating levels of TNF-α in HFD mice. Compared to LFD mice increased circulating levels of TNF-α were already detectable in HFD mice at 24 w, were consistently elevated over time and were associated with increased numbers of renal macrophages (Mϕ).

Mϕ are known to promote deposition of collagen I^14^. Indeed, here Mϕ accumulation strongly correlated with collagen I deposition. Interestingly, however, collagen deposition did not increase proportionally with the marked glomerular and tubulo-interstitial lesions observed by MT staining. Further analysis revealed that obesity promoted the deposition of amyloid, rather than collagen in the kidney in this strain of C57BL/6J mice and prompted us to investigate a potential link between obesity and amyloidosis.

Renal amyloidosis is a rare but severe disease with poor prognosis in which misfolded proteins aggregate to form extracellular fibrillary depositions that destruct the glomerular and tubular architecture^32^,^33^. The initiation and progression of some forms of amyloidosis is driven by proteins involved in chronic inflammatory processes leading to secondary amyloidosis^34^. Here, we found SAA to be a major constituent of the amyloid depositions in kidneys of both LFD and HFD mice. Together with our observations of amyloidosis in other organs, emphasizing the systemic nature of the disease, it may be best classified as AA amyloidosis (AA). Interestingly, systemic SAA levels in HFD mice were increased as well suggesting that obesity, via induction of enhanced SAA production, can provoke or accelerate amyloidogenesis.

SAA is an acute phase protein that preferentially associates with High Density Lipoprotein (HDL)^24^. SAA is thought to enhance HDL’s affinity for cholesterol laden Mϕ^35^, directing the HDL particle to sites of Mϕ accumulation. On reaching the Mϕ, SAA is separated from the HDL particle^36^ which under conditions of chronic Mϕ accumulation could promote an increase in local SAA levels and potentially favor amyloidogenesis. We indeed detected enhanced SAA protein in the HDL fraction of HFD mice. This, in conjunction with chronically increased renal Mϕ in HFD mice, suggests that such a mechanism may have contributed to the development of amyloid deposits.

**Figure 9:**
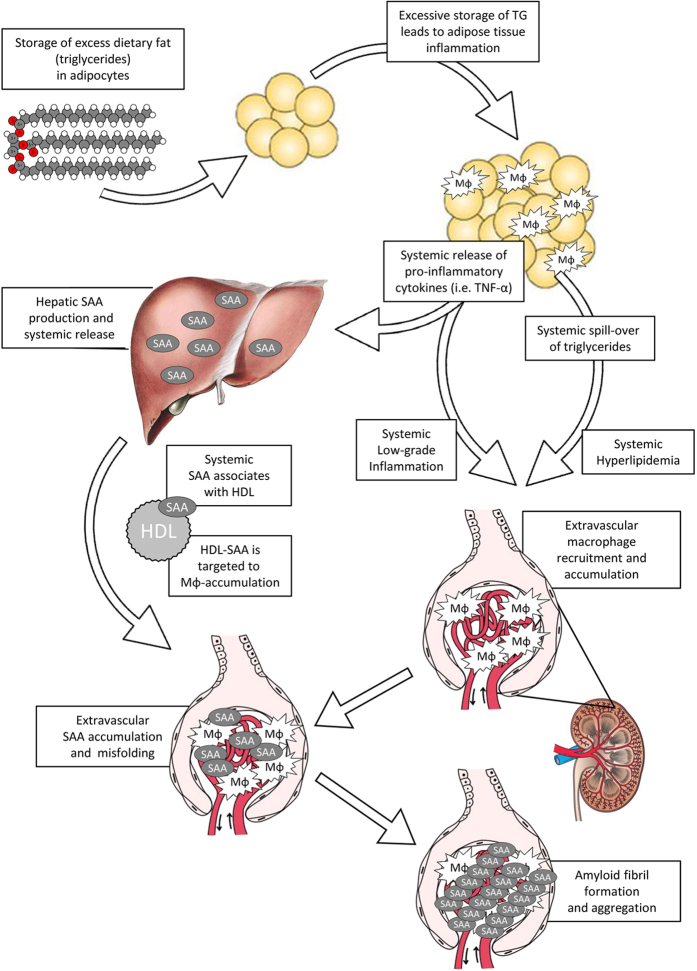
Proposed conceptual link between high-fat diet feeding and renal amyloidosis. HFD-feeding results in an excess of circulating triglycerides which are primarily stored in the adipose tissue (AT). To cope with excess triglycerides the adipocytes increase both in size and number. Eventually, inadequate oxygenation and lipotoxicity will trigger local inflammatory pathways, thereby promoting the recruitment of macrophages (Mϕ) to the AT. Infiltrating Mϕ enhance the local production of pro-inflammatory factors, which eventually enter the circulation and lead to systemic low-grade inflammation (LGI). In parallel, as the AT has insufficient lipid storage capacity, TGs spill over to the circulation leading to systemic hyperlipidemia. Both LGI and hyperlipidemia may promote macrophage recruitment in a variety of vasculatures including the glomerular microvasculature. Systemic AT-derived cytokines, in particular TNF-α, also affect the liver. TNF-α stimulates the production of serum amyloid A (SAA), an acute phase inflammatory reactant which, upon entering the circulation, associates with high density lipoprotein (HDL). Binding of SAA to HDL is thought to target the HDL particle to sites where macrophages accumulate; in this case the glomerular microvasculature. Although it is unclear what happens with SAA once HDL reaches the inflammatory site, it likely dissociates and temporarily resides in the tissue. Therefore, as in HFD-induced obesity chronic inflammation is unresolved, the local concentration of SAA can significantly increase. These high concentrations favor SAA’s amyloidogenic properties, i.e. promote its misfolding and aggregation. As this process progresses amyloid fibrils are formed which destruct the glomerular architecture and eventually impair renal function.

With respect to the source of SAA in obesity, at least two non-mutually exclusive possibilities can be proposed. First, the adipose tissue itself has been identified as a source of SAA in obesity^37^,^38^,^39^,^40^. However, it remains debated whether AT-derived SAA can be secreted. More likely, obesity induced chronic production of pro-inflammatory cytokines, in particular TNF-α, may stimulate hepatocytes to overproduce SAA^41^. Figure 9 summarizes the conceptual axis along which HFD-feeding could lead to amyloid deposits in the glomerular vasculature considering SAA to be of hepatic origin.

To date, little clinical or experimental evidence exists on a causal link between obesity and AA amyloidosis. However, in case reports patients have been described in which morbid obesity was considered the sole remaining cause of renal AA^37^,^42^. In addition, a recent study in genetically obese Ob/Ob mice demonstrated the occurrence of SAA depositions upon injection with amyloid enhancing factor (AEF)^43^, emphasizing the potential of obesity induced SAA to form amyloid.

Chronically elevated SAA levels are a key prerequisite for AA amyloidosis. However, the approximately 3-fold increase in SAA levels observed in HFD mice, which is similar to those reported in obese humans^37^, contrasts markedly with the approximately 100-fold induction of SAA in for example RA patients^44^. Moreover, LFD mice also developed amyloidosis, albeit at a later time point and to a lesser extent, but without an overt increase in circulating SAA levels. This suggests that other factors must be present that favor local accumulation of SAA over time. Recent studies in a mouse model of senile amyloidosis showed endoplasmatic reticulum stress and autophagy to be associated with apoptosis and extracellular deposition of the amyloidogenic ApoA2 lipoprotein^45^. Whereas such mechanisms may be involved in SAA accumulation as well, at present we cannot fully explain the age-dependent development of amyloidosis. Interestingly, background strain and animal supplier may be important determinants as well^19^. This suggests that susceptibility to develop the age-dependent amyloidosis is genetically determined. Comparing the genome of this strain with C57Bl/6 mice from other suppliers offers a possibility to further elucidate the underlying molecular mechanism of this age-dependent renal phenotype, and perhaps its relevance for human CKD.

Our study has several limitations. To minimize confounding factors, we performed this study in mice with strictly defined diets that only differed in fat content. The Western style diet of humans is of course more complex being also high in protein, cholesterol, fructose, and salt. Overabundance of all of these dietary factors has been implicated in the detrimental effects of obesity as well^46^,^47^,^48^,^49^. Moreover, our study was designed as an observational study intended to characterize and monitor the evolution of structural alterations in the kidney in conjunction with renal function and temporal changes in metabolic and inflammatory factors. Since our study did not include specific interventions we can only speculate on potential mechanisms involved based on associations. Despite these limitations, the data presented here demonstrate that obesity-induced systemic inflammation plays a crucial role in the progression of age-dependent renal dysfunction and structural injury. In particular, our study indicates obesity as a potential risk factor for the development of AA amyloidosis at least in susceptible individuals. Future studies on the role of SAA, and its association with the HDL particle in the course of obesity are needed to address its relevance as a risk factor for the progression of age-associated renal amyloidosis.

## Methods

### Mice, diets and housing conditions

Male C57BL/6J (JAX) mice (99 in total) at the age of 6 weeks, weighing 20 ± 2 grams, were obtained from Charles River (Charles River Laboratories, France). Mice were single housed and during the entire experiment kept on a 12 hour light:12 hour dark cycle with ad libitum access to food and water. After arrival all mice received a low-fat control diet (LFD; 10% lard; Research Diets, NJ, USA; D12450HY) for six weeks after which 54 mice switched to a high-fat diet (HFD; 45% lard; Research Diets; D12451). LFD and HFD mice were randomly divided in three groups, receiving either 24, 40 or 52 weeks of LFD control or HFD diet. Bodyweight was determined at regular intervals. To ensure experiments were conducted in a chronic state of obesity, mice that exhibited a >15% weight loss compared to their peak weight, were excluded from all analyses. Six mice were excluded from the study due to the development of dermatitis, that is n = 2 in the HFD24 group, n = 3 in the HFD52 group, and n = 1 in the LFD52 cohort. The experimental study design with final cohort sizes is given in Suppl. Figure 4. All experiments were approved by the Committee on Animal Experimentation of the University of Groningen and were conducted in accordance to the Dutch Law on Animal Experimentation (WOD), following international guidelines on animal experimentation. Certain data, namely body weight development over time and plasma glucose, insulin, cholesterol and triglyceride levels were published previously^31^, but have been included here as well for completeness of the basic phenotypic characterization.

### Blood collection and sacrifice procedure

Prior to sacrifice mice were fasted for 6 hours (8:00–14:00). Mice were anesthetized by isoflurane (2% in oxygen). Aorta and left ventricle hemodynamic measurements were performed using a Millar catheter (Mikro-tip 1.4F; SPR-839, Millar Instruments, Houston, TX, USA) via the right carotid artery in n = 8 mice per experimental group, as described elsewhere^50^. Hereafter, whole blood samples were collected by cardiac puncture, immediately transferred to EDTA coated tubes (Sarstedt) and put on ice. Samples were spun down (5 °C, 3000 rpm) and plasma was isolated and stored at −80 °C. The lower part of the aorta was cut and the circulation was flushed with NaCl. Kidneys, heart (compartmentalized), adipose tissue (mesenteric, gonadal and perirenal depots) were isolated, weighed and partially snap frozen and stored at −80 °C and partially fixed in paraformaldehyde and embedded in paraffin.

### Histology

#### Biochemical staining

Paraffin embedded kidney sections (4 μm), were fixed in aceton and deparaffinized as described elsewhere^51^, and stained for Masson’s trichrome (MT) and periodic acid Schiff’s (PAS). MT and PAS sections were assessed by brightfield microscopy and representative pictures of cortical regions and individual glomeruli were taken at 100× and 400× magnification respectively (Olympus BX50/DP72, Olympus, Shinjuku, Tokio, Japan).

#### Amyloid staining and quantification

Congo red (CR) dye has been acknowledged by the International Society of Amyloidosis as the golden standard for detection of amyloid^52^. CR staining was executed according to internationally accepted staining procedures^53^. Briefly, paraffin sections (8 μm) were deparaffinized and rehydrated as described previously, followed by staining with Mayer’s Hemalum for 15 seconds and destained under running tapwater for 10 minutes. Hereafter sections were first stained for 30 minutes with freshly filtered solution 1 (saturated solution of NaCl (2%) in 80% Ethanol and 0.01% NaOH) followed by staining for 30 minutes with freshly filtered solution 2 (saturated solution of NaCl (2%) and Congo red (1%; Sigma Aldrich) in 80% Ethanol and 0.01% NaOH). Slides were washed twice with Ethanol followed by two washes with demineralized water. Slides were covered with Kaiser’s glycerol gelatin and a coverglass. Sections were assessed by polarized light, with apple-green birefringence confirming the amyloid nature of Congo Red stained tissues.

CR dye was quantified in glomerular and interstitial/tubular regions using Spectral Imaging (Nuance FX, PerkinElmer, Waltham, MA, USA). In short, approximately 6 cortical fields were selected containing a total of 30 glomeruli. The CR specific spectrum was determined and spectral images were transformed to black and white for further quantification of area and intensity using Aperio ImageScope Software (Aperio Technologies, CA, USA). Distinction was made between intra and –extraglomerular areas and data are presented as % CR positive area (amyloid score).

#### Immunohistochemistry

All immunohistochemical stainings were performed on air-dried renal cryo sections (4 μm). Sections were incubated with primary antibodies for rat-anti-mouse CD68 (FA11 clone, Serotec, Puchheim, Germany), rabbit-anti-mouse Collagen I (Abcam, Cambridge, UK) or goat-anti-mouse SAA (R&D systems, Abingdon, UK), combined with secondary unlabeled rabbit-anti-rat for CD68 (Vector laboratories, Burlingame, CA, USA), and rabbit-anti-goat for SAA (Dako, Heverlee, Belgium) antibodies respectively. A commercially available AEC kit, using Anti-Rb HRP polymer was used to visualize protein expression (Dako). Sections were digitalized at 400× magnification using Hamamatsu NDP slide scanner (Nanozoomer 2.0HT, Hamamatsu Photonics, Hamamatsu, Japan) and six matched CD68 and collagen I cortical sections were selected at 200× magnification from approximately 10 mice per experimental group per time point. SAA was only stained on sections from the LFD52 and HFD52 cohort. Staining was quantified using Aperio ImageScope Software (Aperio Technologies).

### Urine collection and analyses

Two weeks prior to sacrifice mice were transferred to metabolic cages for 20h urine collection (12:00 p.m. day 1 until 8:00 a.m. day 2). Mice had ad libitum access to food and water. Samples were collected and spun down at 15.000rpm, urine was transferred and volume was determined. Urinary albumin (Bethyl laboratories, Montgomery, TX, USA), NGAL (R&D systems) and KIM-1 (R&D systems) concentration were determined by ELISA, and adjusted for urinary volume.

### Plasma analyses

Plasma samples were collected as detailed above. All analyses were performed using commercially available kits and according to manufacturer’s instructions unless stated otherwise. Plasma leptin and adiponectin were determined by ELISA (R&D systems), cholesterol and triglycerides by biochemical assays (cat.no. 11489437 and 11488872 respectively Roche Diagnostics, Almere, The Netherlands). Glucose was determined using a hand held glucometer (LifeScan, Milpitas, CA, USA) directly after blood was drawn. Insulin was determined by ELISA (Alpco, Tilburg, The Netherlands). Blood urea nitrate was determined by biochemical assay (QuantiChrom Urea Assay Kit, BioAssaySystems, Hayward, CA, USA). Inflammatory markers TNF-α, IL-6, IL-1β were measured as part of a Mesoscale discovery 10-plex multispot Mouse cytokine assay (Gaithersburg, USA). Mouse plasma MCP-1 (Thermo-Fisher scientific, Pittsburgh, PA) and mouse Serum Amyloid A protein (Tridelta, Maynooth, Ireland) were determined by ELISA.

### RNA isolation and RT-qPCR

RNA was isolated from thirty 5μm thin cryo sectons using Rneasy Mini plus Kit (Qiagen, Westburg, Leusden, The Netherlands) according to the manufacturer’s instruction. Procedures for determining RNA integrity, quality and quantity as well as cDNA synthesis can be found elsewhere^51^. Expression of selected genes was performed by Assays-On-Demand primer probe sets (*Tnf*; Mm00443258_m1, *Mcp-1*; Mm00441242_m1, IL-6; Mm00446190_m1, IL-1β; Mm00434228_m1, Ppia; Mm02342430_g1, AB Systems, Foster City, CA). Data is expressed as 2^−ΔCt^.

### FPLC and Western blot

Pooled plasma samples for LFD52 and HFD52 were subjected to fast protein liquid chromatography (FPLC) gel filtration using a Superose 6 column (GE Healthcare, Hoevelaken, The Netherlands) as described elsewhere^54^. Fractions corresponding to HDL were pooled to assess SAA protein concentration. Protein concentrations of pooled samples were measured using BCA assay (Pierce). Equal amounts of protein were separated by electrophoresis (Beckman Instruments, Brea, CA, USA) and blotted onto nitrocellulose membrane. After blocking, membranes were incubated with primary mouse SAA antibody (R&D systems) followed by incubation with appropriate secondary antibodies.

### Statistical analyses

Data were analyzed using GraphPad Prism (version 5.00, GraphPad Software, San Diego, CA, USA). Non-parametric Mann-Whitney U tests were performed for comparing LFD and HFD groups within a time point, and Kruskall Wallis ANOVA with Dunn’s post hoc test was used to determine differences between time points within the designated diet. Correlation analyses were performed using Spearman r. p-values lower than 0.05 were considered significant. Data are expressed as mean ± standard error of the mean (SEM). Significance level was set at p < 0.05.

## Additional Information

**How to cite this article**: van der Heijden, R. A. *et al.* Obesity-induced chronic inflammation in high fat diet challenged C57BL/6J mice is associated with acceleration of age-dependent renal amyloidosis. *Sci. Rep.* **5**, 16474; doi: 10.1038/srep16474 (2015).

## Supplementary Material

Supplementary Information
