# Supplementary material for: Obesity-induced chronic inflammation in high fat diet challenged C57BL/6J mice is associated with acceleration of age-dependent renal amyloidosis
Source: Sci Rep. 2015 Nov 13;5:16474. doi: 10.1038/srep16474 (PMC4643235; doi:10.1038/srep16474)
Supplement: Supplementary Information [file srep16474-s1.pdf]

Supplementary data to:

# Obesity-induced chronic inflammation in high fat diet challenged C57BL/6J mice is associated with acceleration of age-dependent renal amyloidosis

by

Roel A. van der Heijden, Johan Bijzet, Wouter C. Meijers, Gopala K. Yakala,

Robert Kleemann, Tri Q. Nguyen, Rudolf A. de Boer, Casper G. Schalkwijk, Bouke P.C. Hazenberg,

Uwe J. F. Tietge and Peter Heeringa

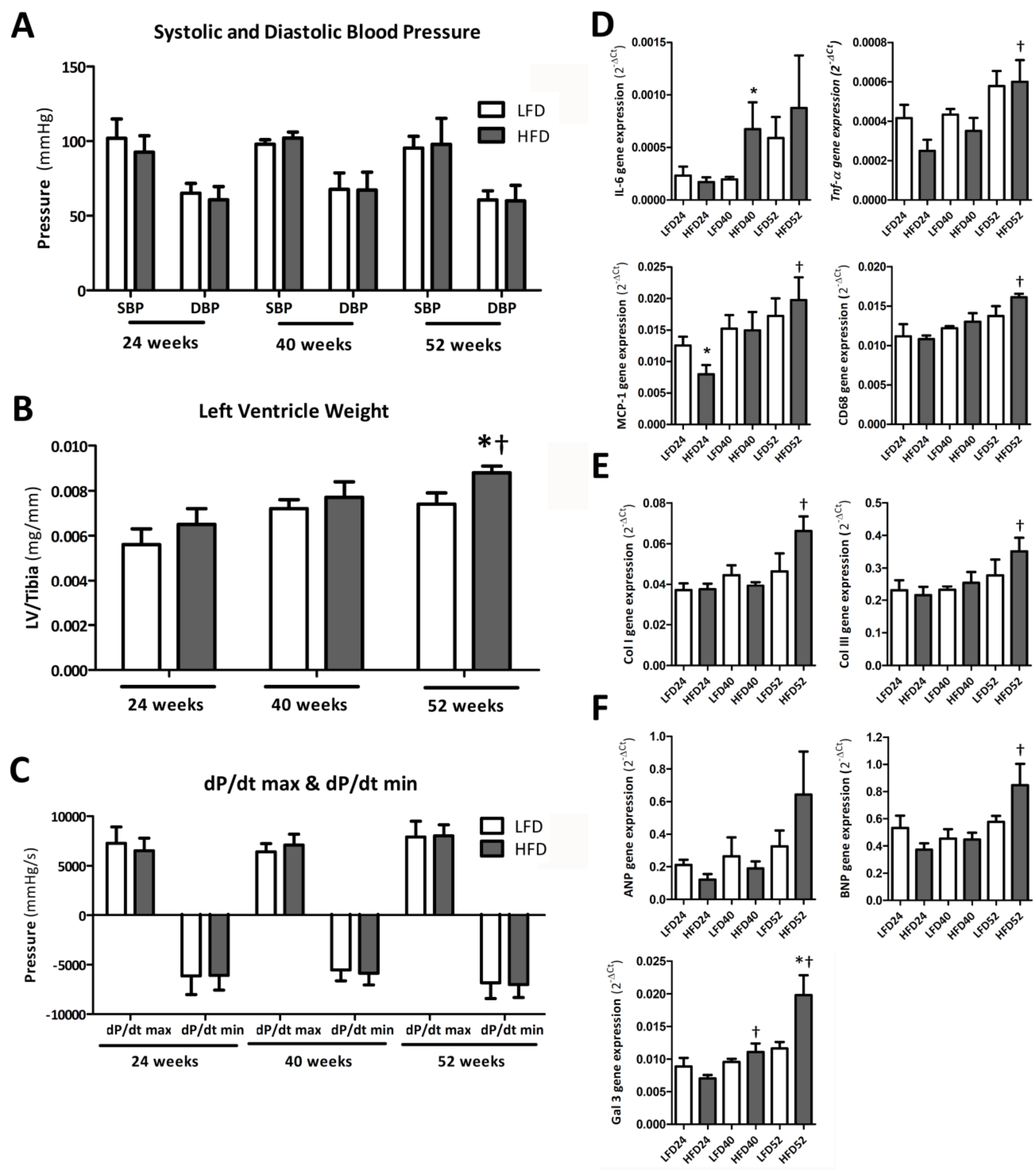


**Supplementary Figure S1. Left ventricle hemodynamic and gene expression parameters**. (A) Left ventricle (LV) systolic and diastolic blood pressure (SBP and DBP). (B) LV weight corrected for tibia length. (C) Ventricular contractility. Expression of (D) inflammatory, (E) fibrotic and (F) injury genes in LV. Data expressed as mean ±SEM. Significance level set at $p<0.05$. *=significantly different from LFD same time point, ‡=significantly different from same diet 40w.

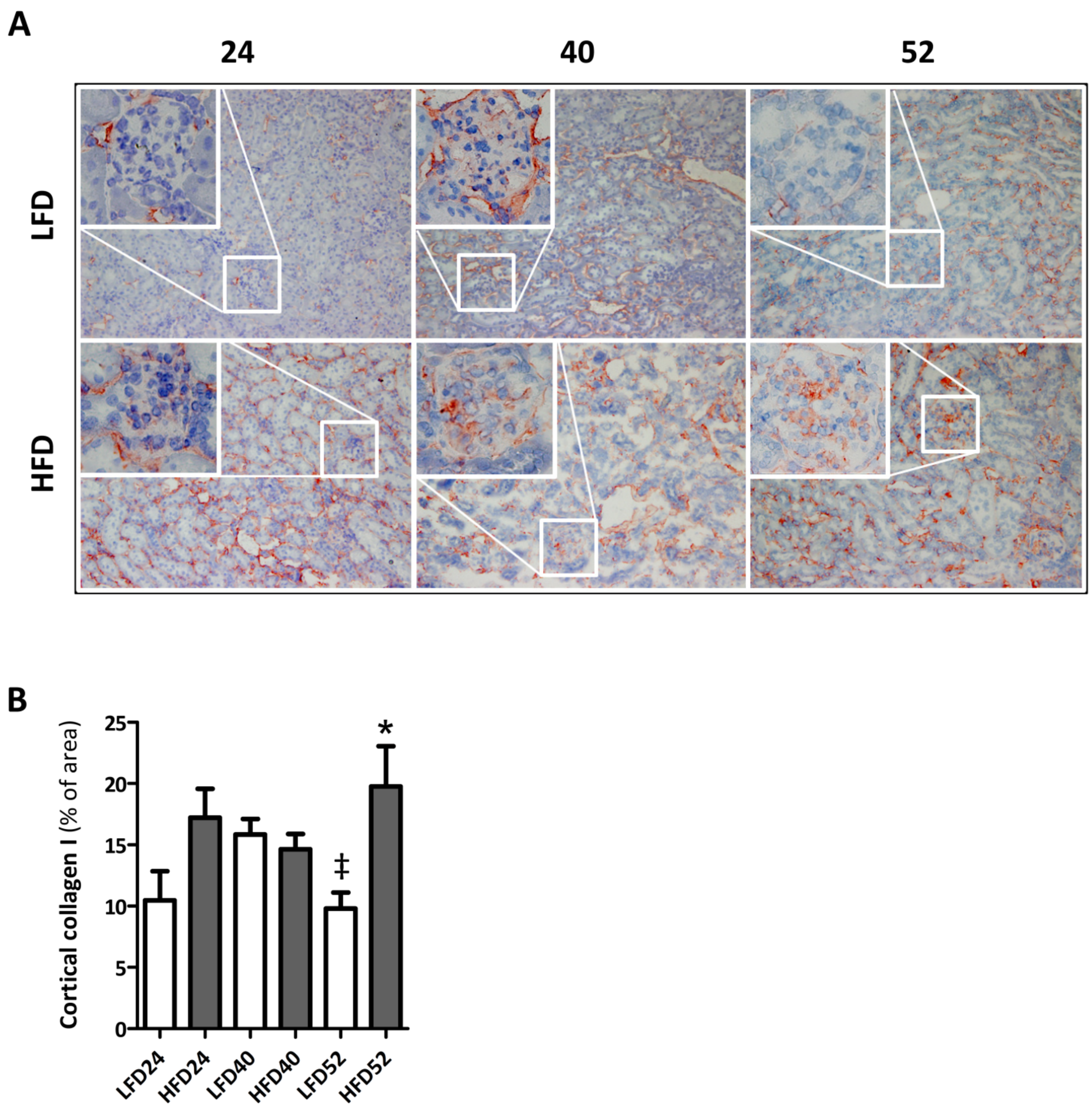


**Supplementary Figure S2. Cortical collagen I protein expression with aging and HFD-feeding.** (A) Representative overviews of the renal cortex (100x) and individual glomeruli (inset; 400x) from collagen I stained sections. (B) Quantification of staining shows significantly elevated collagen I protein in HFD groups at 24 and 52 weeks, but no significant change over time for HFD mice. Data expressed as mean ±SEM. Significance level set at $p<0.05$. *=significantly different from LFD same time point, ‡=significantly different from same diet 40w.

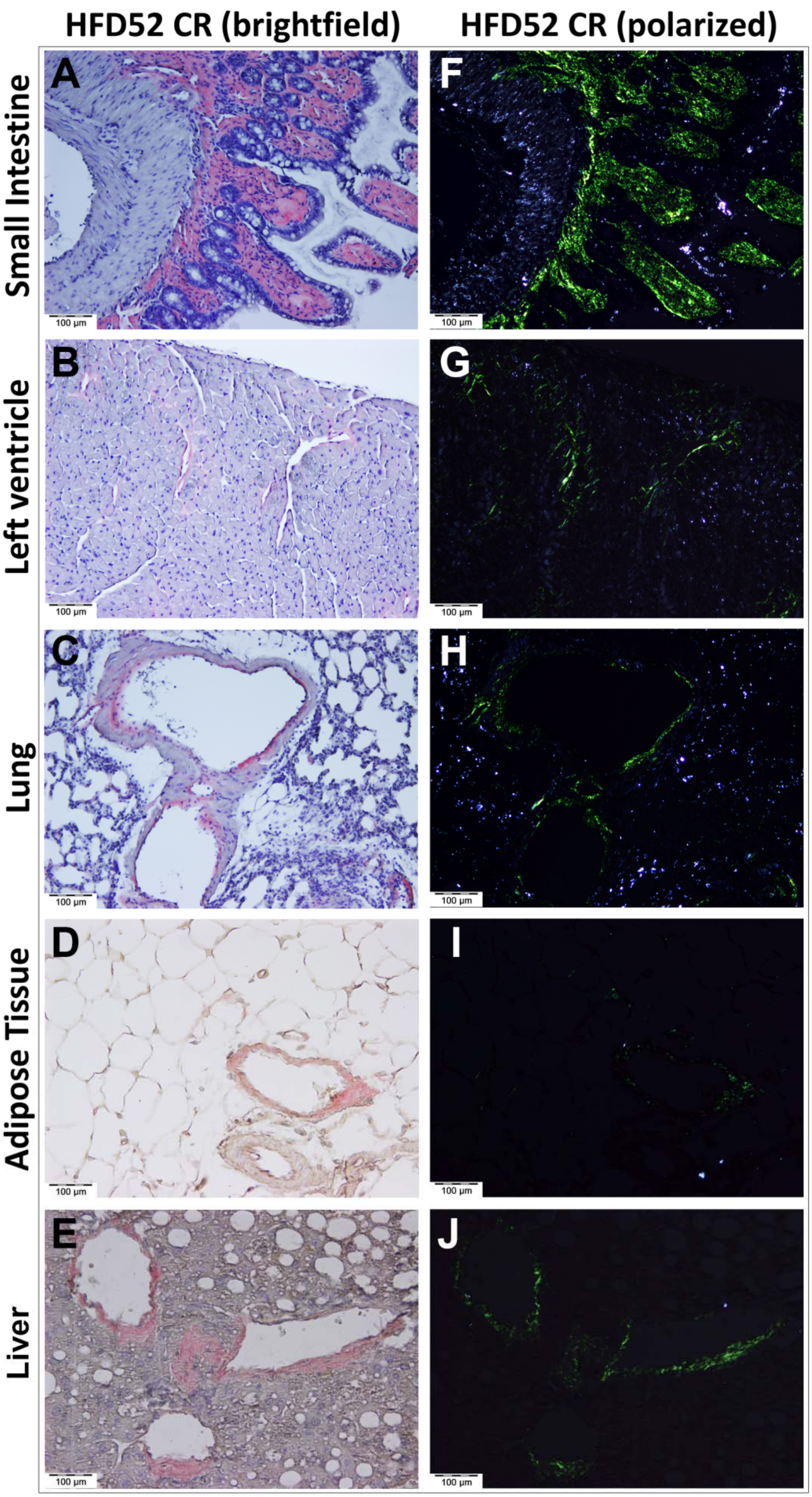


**Supplementary Figure S3.** Congo Red staining of (A) small intestine, (B) lung, (C) left ventricle, (D) adipose and (E) hepatic tissue sections, assessed under brightfield (A-E) and polarized light (F-J) showing deposition of amyloid fibrils located predominantly in the vasculature (100x magnification).

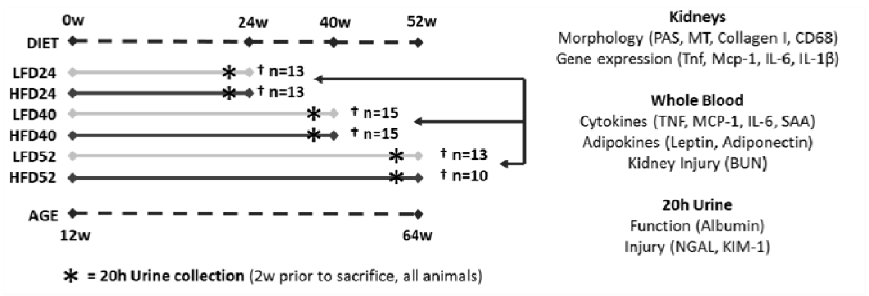


**Supplementary Figure S4.** Experimental design.
